# ﻿Three new species of Theridiidae Sundevall, 1833 (Araneae) from Xizang, China

**DOI:** 10.3897/zookeys.1251.164206

**Published:** 2025-09-03

**Authors:** Jiahui Gan, Xiaoqi Mi, Cheng Wang, Liqing Fan

**Affiliations:** 1 Guizhou Provincial Key Laboratory for Biodiversity Conservation and Utilization in the Fanjing Mountain Region, Tongren University, Tongren, 554300 Guizhou, China Tongren University Tongren China; 2 Key Laboratory of Forest Ecology in Xizang Plateau of the Ministry of Education, Institute of Plateau Ecology, Xizang Agricultural & Animal Husbandry University, Linzhi, 860000 Xizang, China Xizang Agricultural & Animal Husbandry University Linzhi China

**Keywords:** Cob-web spider, DNA barcodes, morphology, Southwestern China, taxonomy

## Abstract

Three new species belonging to the spider family Theridiidae are described based on materials collected from Xizang Autonomous Region, Southwestern China: *Moneta linzhi* Gan, Mi & Wang, **sp. nov.** (♀♂), *M. yinae* Gan, Mi & Wang, **sp. nov.** (♀♂) and *Phoroncidia cibagou* Gan, Mi & Wang, **sp. nov.** (♀♂). Diagnostic photos of the habitus and copulatory organs, and a distribution map are provided.

## ﻿Introduction

Theridiidae Sundevall, 1833, a species-rich spider family, currently comprises 2596 extant species in 133 genera from around the world (WSC 2025). It has a high diversity in China, and currently, at least 447 extant species in 61 genera are recorded (WSC 2025). However, the taxonomic study of the Chinese theridiids remains unsatisfactory because more than one-third of its species are known only from a single sex. A most influential revision of the Chinese theridiids was provided by Zhu (1998), who described 223 species in 27 genera, including 87 new to science and 33 new recorded to China.

Xizang is located in southwest China, and currently known by 26 theridiid species belonging to 12 genera, of which 11 are endemics, and five are known only from a single sex (WSC 2025). The taxonomic study of the theridiid from Xizang was nearly halted after Hu (2001), who described seven endemic species, and only one new species has been described by Lin et al. (2024a).

Over the last two years, spider surveys in two National Nature Reserves from Linzhi City, Xizang, China, were carried out. After the examination of theridiid specimens, three species belonging to two genera are recognized as new to science. The goal of the present work is to describe those of three species.

## ﻿Materials and methods

Specimens were collected by beating shrubs or hand collecting and preserved in 90% ethanol. Specimens are deposited in the Museum of Tongren University (TRU) in Tongren, China. They were examined using an Olympus SZX 16 stereomicroscope. After dissection, the vulvae were cleared in trypsin enzyme solution before examination and imaging. Left male palps were used for the descriptions and illustrations. Photographs of the copulatory organs and habitus were taken with a Kuy Nice CCD camera mounted on an Olympus BX43 compound microscope. Compound focus images were generated using Helicon Focus v. 6.7.1. ArcGIS v.10.4 software was used to create a distribution map.

A partial fragment of the mitochondrial cytochrome *c* oxidase subunit I (COI) gene of the three species was amplified and sequenced using the primers CO1-TY-F1 and CO1-TY-R1 (Yamasaki et al. 2018). The accession numbers are provided in Table 1.

**Table 1.** Voucher specimen information.

Species	Voucher code	Sex	GenBank accession number
*Moneta linzhi* Gan, Mi & Wang, sp. nov.	TRU-XZ-THR-0004	♀	PX021805
	TRU-XZ-THR-0005	♀	PX021807
	TRU-XZ-THR-0006	♂	PX021809
*M. yinae* Gan, Mi & Wang, sp. nov.	TRU-XZ-THR-0030	♀	PX021802
	TRU-XZ-THR-0031	♀	PX021804
	TRU-XZ-THR-0060	♂	PX021803
	TRU-XZ-THR-0061	♂	PX021806
*Phoroncidia cibagou* Gan, Mi & Wang, sp. nov.	TRU-XZ-THR-0072	♀	PX021808
	TRU-XZ-THR-0073	♂	PX021810

All measurements are given in millimetres. Leg measurements are given as total length (femur, patella + tibia, metatarsus, tarsus). References to figures in the cited papers are listed in lowercase type (fig. or figs), and figures in this paper are noted with an initial capital (Fig. or Figs).

Abbreviations used in the text and figures are as follows:

**AER** anterior eye row;

**ALE** anterior lateral eye;

**AME** anterior median eye;

**BP** basal epigynal plate;

**C** conductor;

**CD** copulatory duct;

**Chk** cymbial hook;

**CO** copulatory opening;

**E** embolus;

**EA** embolic apophysis;

**EB** embolic base;

**FD** fertilization duct;

**H** hood;

**PER** posterior eye row;

**PLE** posterior lateral eye;

**PME** posterior median eye;

**PTA** prolateral tegular apophysis;

**S** spermatheca;

**ST** subtegulum;

**VTA** ventral tegular apophysis.

## ﻿Taxonomy

### ﻿Family Theridiidae Sundevall, 1833

#### ﻿Genus Moneta

Taxon classificationAnimaliaAraneaeTheridiidae

O. Pickard-Cambridge, 1871

4FFC7B61-36FF-5585-8A77-D3A7C4D4DA47

##### Type species.

*Moneta spinigera* O. Pickard-Cambridge, 1871.

##### Notes.

*Moneta* is represented by 22 species, mainly distributed in East and Southeast Asia, and extending to Australia (WSC 2025). Of these, 13 are known from China, and 10 are endemics (WSC 2025). The genus is considered to be related to *Episinus* Latreille, 1809, from which it can be distinguished from the latter by the clypeus extending far in front of eyes; eye region almost straight laterally; abdomen elongated with two humps, truncated anteriorly, and not overhanging the carapace; and the male palp usually with a lateromarginal projection on the cymbium (Vanuytven 2021). The taxonomy of the genus remains poorly studied, as 10 species are known only from a single sex, and several others cannot be reliably identified due to the absence of diagnostic illustrations or photographs (WSC 2025). The genus was not previously reported from Xizang, and the species described below represents its westernmost known record.

#### ﻿ Moneta linzhi

Taxon classificationAnimaliaAraneaeTheridiidae

Gan, Mi & Wang sp. nov.

DC8D544A-F035-5EA0-BEC9-B045026F64FB

https://zoobank.org/D242E3EE-8985-4850-AA43-829032A6773F

Figs 1, 2, 5A, B, 8

##### Type material.

***Holotype*** • ♂ (TRU-XZ-THR-0001), China: Xizang Autonomous Region, Linzhi City, Bomi County, Gangyunshanlin Scenic Area (29°52.66'N, 95°34.22'E, c. 2658 m), 21.V. 2024, X.Q. Mi et al. leg. ***Paratypes*** • 4♀13♂ (TRU-XZ-THR-0002–0018), same data as for holotype; • 2♀ (TRU-XZ-THR-0019–0020), same site as for holotype, 29.VI. 2023, C. Wang leg.

##### Etymology.

The species name is a noun in apposition and derived from the type locality, Linzhi City.

##### Diagnosis.

**Figure 1.**
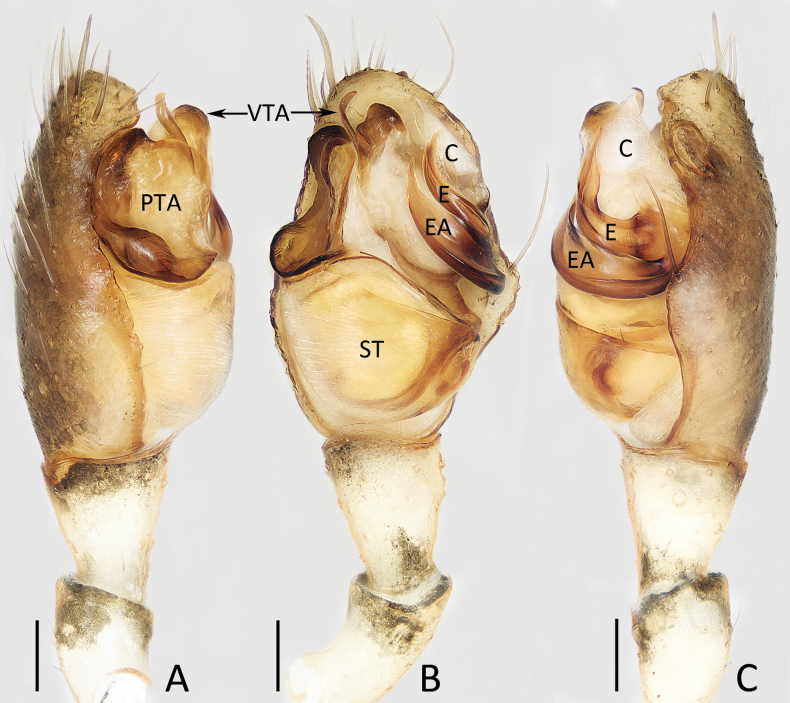
Male palp of *Moneta linzhi* Gan, Mi & Wang, sp. nov., holotype. A. Prolateral view; B. Ventral view; C. Retrolateral view. Scale bars: 0.1 mm.

**Figure 2.**
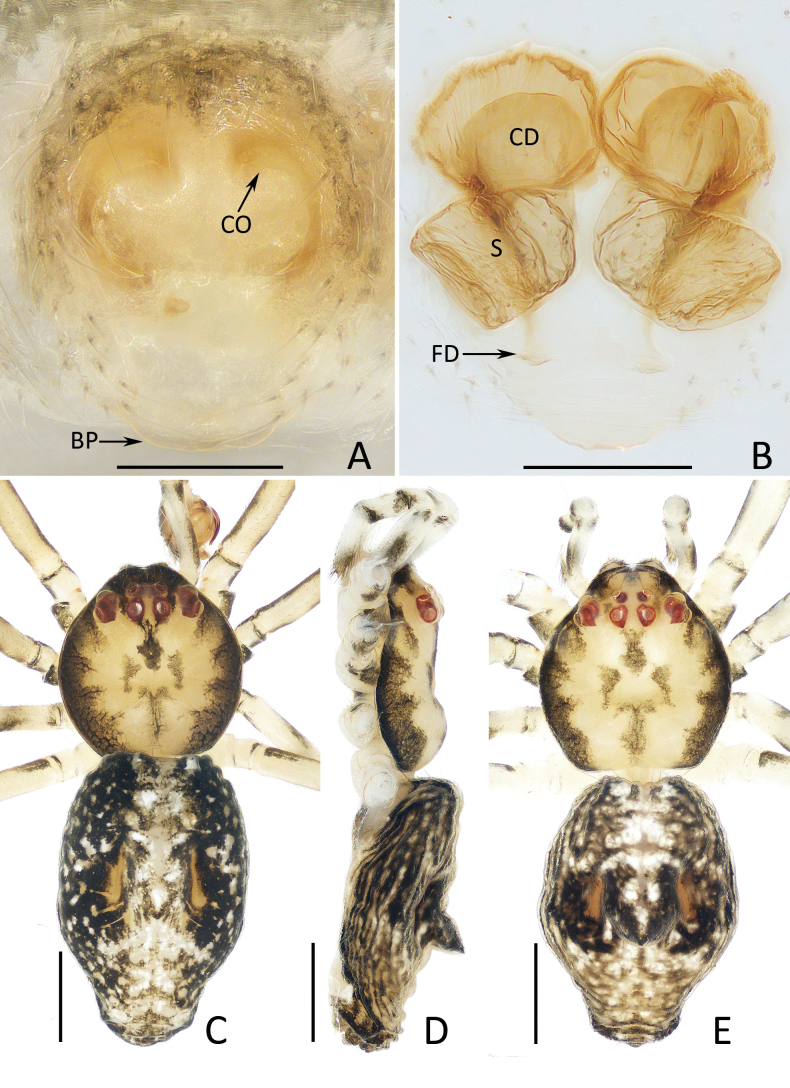
*Moneta linzhi* Gan, Mi & Wang, sp. nov. A, B, D, E. Female paratype (TRU-XZ-THR-0002), C. Male holotype. A. Epigyne, ventral view; B. Vulva, dorsal view; C, E. Habitus, dorsal view; D. Ditto, lateral view. Scale bars: 0.1 mm (A, B); 0.5 mm (C–E).

The male of this species is closely similar to that of *M. tumulicola* Zhu, 1998 in having a very similar palpal structure, but it can be easily distinguished by the following: (1) embolic apophysis (EA) is sheet-shaped in ventral view (Fig. 1B) vs almost bar-shaped (Zhu 1998: fig. 190A); and (2) ventrally extending portion of the distal cymbium is about 2/5 the bulb width and having a blunt end in prolateral view (Fig. 1A) vs about 4/5 the bulb width and having a rather pointed end (Zhu 1998: fig. 190B). This species is also similar to that of *M. oupeng* Lin & Li, 2024 in having similar copulatory organs, but it can be easily distinguished by the following: (1) embolus (E) is arc-shaped in ventral view (Fig. 1B) vs invisible (Lin et al. 2024b: fig. 10B); (2) retrolateral branch of the ventral tegular apophysis (VTA) is sclerotized distally and has a blunt tip in ventral view (Fig. 1B) vs membranous and has a somewhat pointed tip (Lin et al. 2024b: fig. 10B); (3) prolateral tegular apophysis (PTA) is about as long as wide (Fig. 1A) vs obviously wider than long (Lin et al. 2024b: fig. 10A); (4) basal epigynal plate (BP) is far away from the atrium about the atrial length (Figs 2A, 5A) vs almost touched (Lin et al. 2024b: fig. 11A); and (5) copulatory ducts (CD) are obvious wider than long (Figs 2B, 5B) vs almost as wide as long (Lin et al. 2024b: fig. 11B).

##### Description.

**Male** (holotype; Figs 1, 2C). Total length 2.65. Carapace 1.10 long, 1.00 wide. Abdomen 1.61 long, 1.03 wide. Carapace oval, yellow except surrounding dark brown, with irregular dark patch behind PMEs. Radial grooves indistinct. Clypeus projecting, 0.13 high. AER slightly recurved and PER straight. Eye sizes and inter-distances: AME 0.08, ALE 0.11, PME 0.08, PLE 0.08, AME–AME 0.17, AME–ALE 0.17, PME–PME 0.17, PME–PLE 0.21, ALE–PLE 0.17. Chelicerae saffron yellow, base black grey. Endites beige. Labium pale grey, base black grey. Sternum pale yellow and edge black grey. Legs beige, with black grey stripes on side. Measurements of legs: I 5.86 (2.08, 1.75, 1.75, 0.28), II 4.11 (1.28, 1.50, 1.13, 0.20), III 2.22 (0.73, 0.63, 0.63, 0.23), IV 4.33 (1.50, 1.25, 1.25, 0.33). Dorsum of abdomen mainly dark except central portion pale, covered with off-white spots, with central furcella, and pair of longitudinal yellow sigilla lateral to furcella; venter off-white, anterior black grey.

***Palp*** (Fig. 1A–C): femur c. 4.5 times longer than wide, yellow except base and terminus dark prolaterally; patella c. 2 times longer than wide, yellow except terminal dark; tibia c. 1.4 times longer than wide, gradually widened from base to distal end; cymbium almost 1.5 times longer than wide; prolateral tegular apophysis (PTA) irregular, large, mainly sheet-shaped in prolateral view; ventral tegular apophysis (VTA) divided into slender, distally curved prolateral branch and wider, distally sclerotized retrolateral branch; conductor (C) almost triangular, membranous; embolus (E) strongly curved medially, originates from anterior 1/3 portion of retrolateral side of bulb, rather pointed apically; embolic apophysis (EA) sheet-shaped, almost parallel to emblolus, with pointed tip.

**Female** (TRU-XZ-THR-0002, Fig. 2A, B, D, E; TRU-XZ-THR-0003, Fig. 5A, B). Total length 2.39. Carapace 1.01 long, 0.95 wide. Abdomen 1.34 long, 1.00 wide. Clypeus 0.13 high. Eye sizes and inter-distances: AME 0.05, ALE 0.08, PME 0.06, PLE 0.07, AME–AME 0.15, AME–ALE 0.16, PME–PME 0.16, PME–PLE 0.21, ALE–PLE 0.12. Measurements of legs: I 4.99 (1.73, 1.55, 1.48, 0.23), II 3.61 (1.13, 1.15, 1.15, 0.18), III 2.06 (0.65, 0.68, 0.55, 0.18), IV 4.11 (1.38, 1.23, 1.25, 0.25). Habitus (Fig. 2D, E) similar to that of male.

***Epigyne*** (Figs 2A, B, 5A, B): slightly longer than wide, with base plate slightly beyond epigastric groove; atrium oval, centrally located; copulatory openings (CO) beneath antero-lateral portions of atrium; copulatory ducts (CD) oval; spermathecae (S) oblong, slightly less than 2 times wider than long, slightly separated from each other; fertilization ducts (FD) posterior to spermathecae.

##### Distribution.

Known only from the type locality in Xizang, China (Fig. 8).

#### ﻿ Moneta yinae

Taxon classificationAnimaliaAraneaeTheridiidae

Gan, Mi & Wang sp. nov.

4CCFBC70-A53D-533B-9AF7-834318EC8FCF

https://zoobank.org/1DEDFCA5-8FA3-49C6-A568-E5E4122159CB

Figs 3, 4, 8

##### Type material.

***Holotype*** • ♂ (TRU-XZ-THR-0021), China: Xizang Autonomous Region, Linzhi City, Chayu County, Cibagou National Nature Reserve (28°36.03'N, 97°4.01'E, c. 2200 m), 22–27.VI. 2023, C. Wang leg. ***Paratypes*** • 29♀12♂ (TRU-XZ-THR-0022–0062), same data as for holotype; • 1♀1♂ (TRU-XZ-THR-0063–0064), Motuo County, Beibeng Township, De'ergong Village (29°10.84'N, 95°8.67'E, c. 2200 m), 25.V. 2024, X.Q. Mi et al. leg.

##### Etymology.

The epithet is a patronym in honor of Prof. Changming Yin (late), who contributed to the taxonomy of Chinese spiders; noun in genitive case.

##### Diagnosis.

**Figure 3.**
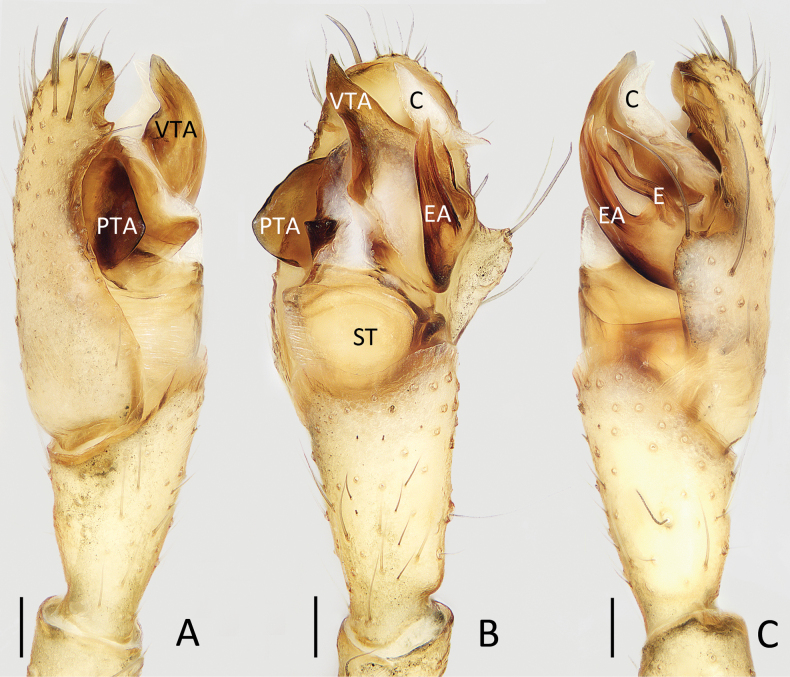
Male palp of *Moneta yinae* Gan, Mi & Wang, sp. nov., holotype. A. Prolateral view; B. Ventral view; C. Retrolateral view. Scale bars: 0.1 mm.

**Figure 4.**
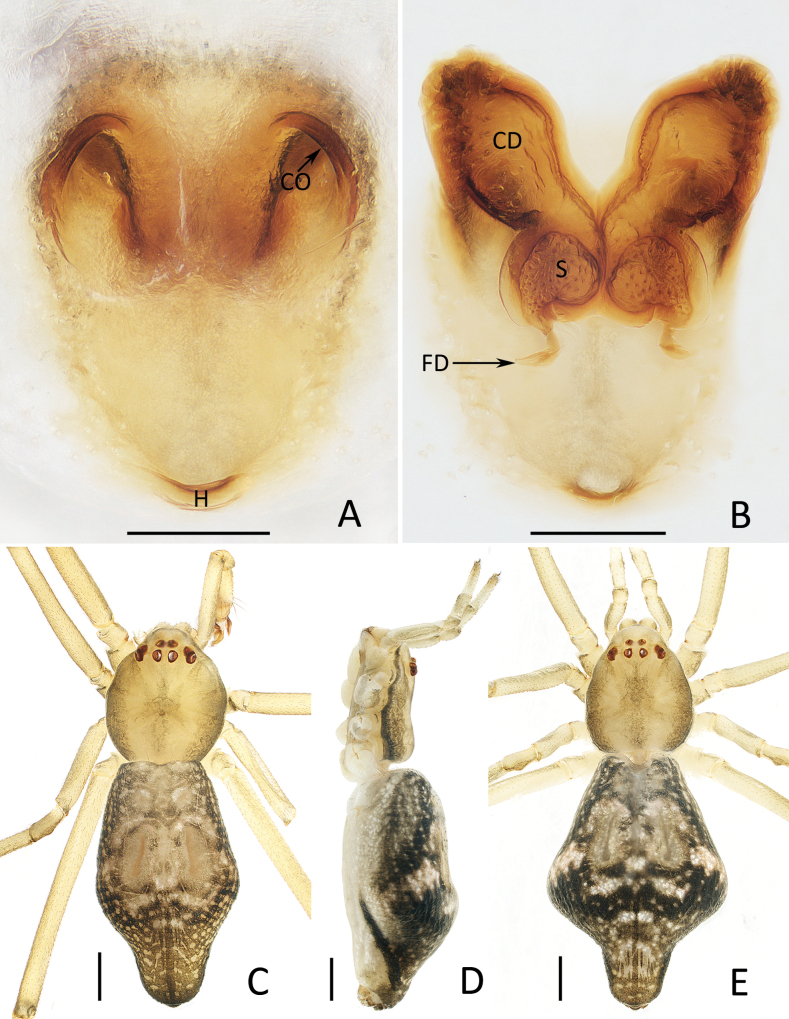
*Moneta yinae* Gan, Mi & Wang, sp. nov. A, B, D, E. Female paratype (TRU-XZ-THR-0022), C. Male holotype. A. Epigyne, ventral view; B. Vulva, dorsal view; C, E. Habitus, dorsal view; D. Ditto, lateral view. Scale bars: 0.1 mm (A, B); 0.5 mm (C–E).

This species is similar to that of *M. mirabilis* (Bösenberg & Strand, 1906), but it can be easily distinguished by the following: (1) prolateral tegular apophysis (PTA) is about triangular in ventral view (Fig. 3B) vs crescent-shaped (Zhu 1998: fig. 189C); (2) posterior portion of the ventral tegular apophysis (VTA) is almost triangular in ventral view (Fig. 3B) vs hooked (Zhu 1998: fig. 189C); (3) embolic apophysis (EA) is almost straight in ventral view (Fig. 3B) vs obvious arc-shaped (Zhu 1998: fig. 189C); and (4) epigynal hood is distinct, posteriorly located (Fig. 4A) vs invisible (Zhu 1998: fig. 189B).

##### Description.

**Male** (holotype; Figs 3, 4C). Total length 3.91. Carapace 1.46 long, 1.24 wide. Abdomen 2.57 long, 1.50 wide. Carapace ovoid, beige and edge grey, radial grooves indistinct. Clypeus projecting, 0.18 high. AER slightly recurved and PER straight. Eye sizes and inter-distances: AME 0.09, ALE 0.12, PME 0.10, PLE 0.13, AME–AME 0.18, AME–ALE 0.19, PME–PME 0.18, PME–PLE 0.22, ALE–PLE 0.13. Chelicerae pale yellow. Endite and sternum beige. Labium fulvous, base black grey. Legs pale yellow, without spots. Measurements of legs: I 10.97 (3.80, 3.43, 3.37, 0.37), II 6.96 (2.23, 2.33, 2.17, 0.23), III 3.77 (1.20, 1.17, 1.13, 0.27), IV 8.46 (2.80, 2.43, 2.83, 0.40). Abdomen rhomboid, dorsum grey, with ecru irregular spots, and pair of median sigilla running parallel to abdomen; venter creamy yellow.

***Palp*** (Fig. 3A–C): femur, patella and tibia yellow, femur c. 5.5 times longer than wide, patella and tibia about 2 times longer than wide; cymbium almost 2 times longer than wide; prolateral tegular apophysis (PTA) sub-triangular in ventral view; ventral tegular apophysis (VTA) flat, wider than embolus, base wide and terminal sharp in ventral view; conductor (C) membranous, fusiform in ventral view; embolus (E) slightly curved into wave-shape, originates from retrolateral side of bulb, with base, knife-shaped apophysis extends along embolus, and wider than embolus.

**Female** (TRU-XZ-THR-0022; Fig. 4A, B, D, E). Total length 4.36. Carapace 1.43 long, 1.19 wide. Abdomen 2.93 long, 2.05 wide. Clypeus 0.21 high. Eye sizes and inter-distances: AME 0.07, ALE 0.09, PME 0.08, PLE 0.09, AME–AME 0.18, AME–ALE 0.20, PME–PME 0.18, PME–PLE 0.23, ALE–PLE 0.12. Measurements of legs: I 9.48 (3.17, 2.87, 3.07, 0.37), II 6.19 (2.00, 2.03, 1.93, 0.23), III 3.49 (1.13, 1.13, 1.00, 0.23), IV 7.94 (2.57, 2.17, 2.77, 0.43). Habitus (Fig. 4D, E) similar to that of male, except darker.

***Epigyne*** (Fig. 4A, B): almost 1.5 times longer than wide, posteriorly with hood (H) opened upward; atrium large, nearly heart-shaped, with pair of anterolateral arc-shaped ridges; copulatory openings (CO) invisible, located anterolaterally on atrium; copulatory ducts (CD) anteriorly extending at origin and then turn back and forming bowling ball-like portions; spermathecae (S) almost spherical, touched; fertilization ducts (FD) posterior to spermathecae.

##### Distribution.

Known only from the type locality in Xizang, China (Fig. 8).

#### ﻿Genus Phoroncidia

Taxon classificationAnimaliaAraneaeTheridiidae

Westwood, 1835

30F54C34-F67B-5C31-A69E-90786AFA354D

##### Type species.

*Phoroncidia aculeata* Westwood, 1835.

##### Notes.

*Phoroncidia* is represented by a group of spiders characterised by the eye region strongly overhanging the carapace, and by having a large sclerotized ring around the spinnerets (Gao and Li 2014). It contains 83 species distributed worldwide (WSC 2025). Among the species, 53 (nearly 60%) are known only from a single sex, and at least 20 species cannot be acutely identified due to a lack of enough diagnostic drawings or photos. Currently, nine species, including five endemics, are known from China (WSC 2025).

#### ﻿ Phoroncidia cibagou

Taxon classificationAnimaliaAraneaeTheridiidae

Gan, Mi & Wang sp. nov.

84012E73-EF65-518C-9FB3-E9E4B3D5F7F2

https://zoobank.org/9C430AF8-349C-4AF6-BD0A-B37572159EE5

Figs 5C, D, 6, 7, 8

##### Type material.

***Holotype*** • ♂ (TRU-XZ-THR-0065), China: Xizang Autonomous Region, Linzhi City, Chayu County, Cibagou National Nature Reserve (28°36.03'N, 97°4.01'E, c. 2200 m), 22–27.VI. 2023, C. Wang leg. ***Paratypes*** • 7♀3♂ (TRU-XZ-THR-0066–0075), same data as for holotype.

##### Etymology.

The specific name is a noun in apposition and refers to the type of locality, Cibagou National Nature Reserve.

##### Diagnosis.

**Figure 5.**
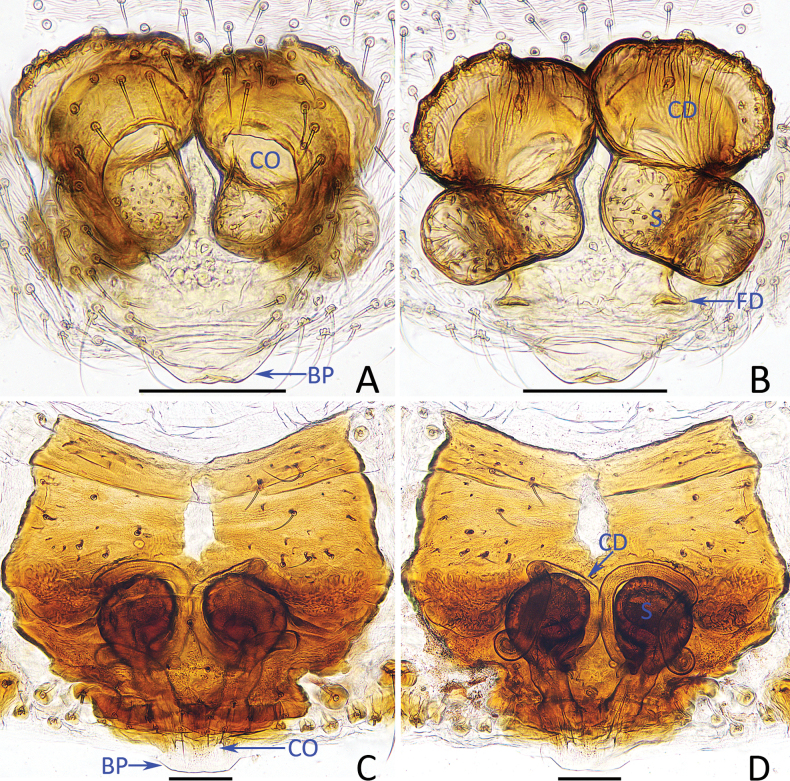
Epigyne of *Moneta linzhi* Gan, Mi & Wang, sp. nov. (A, B) female paratype (TRU-XZ-THR-0003) and *Phoroncidia cibagou* Gan, Mi & Wang, sp. nov. (C, D) female paratype (TRU-XZ-THR-0067). A, C. Epigyne, ventral view; B, D. Vulva, dorsal view. Scale bars: 0.1 mm.

**Figure 6.**
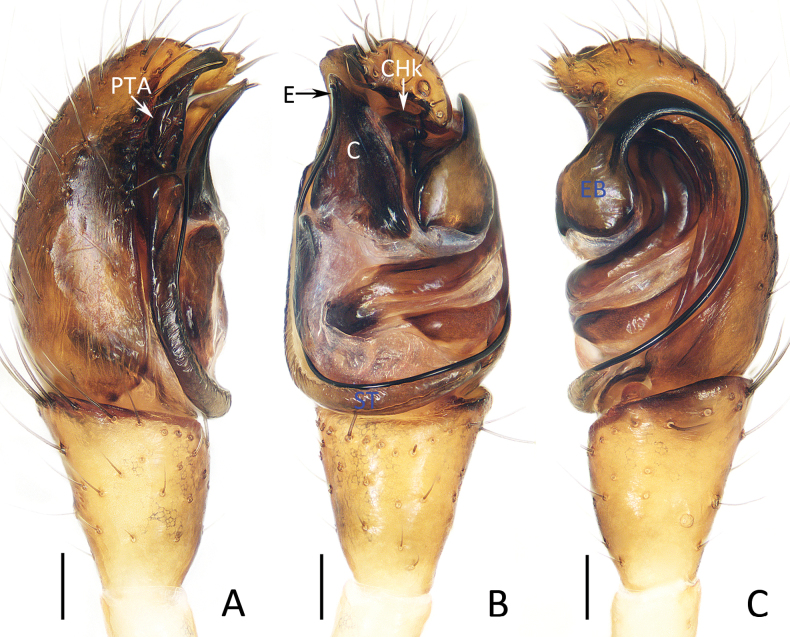
Male palp of *Phoroncidia cibagou* Gan, Mi & Wang, sp. nov., holotype. A. Prolateral view; B. Ventral view; C. Retrolateral view. Scale bars: 0.1 mm.

**Figure 7.**
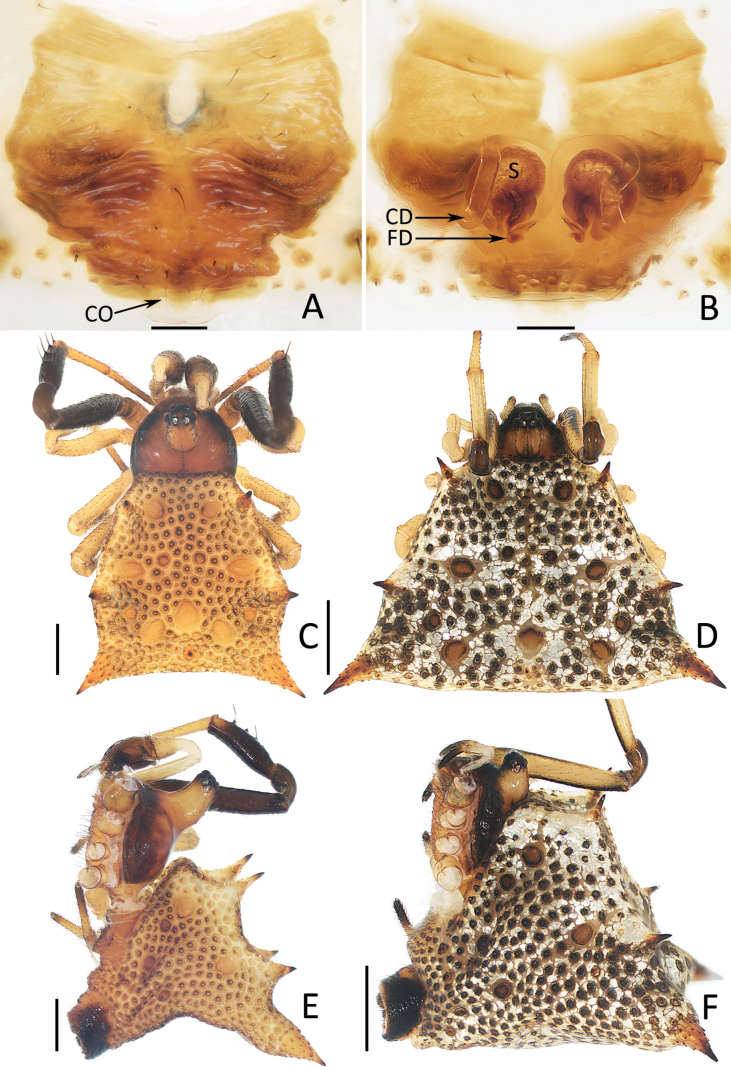
*Phoroncidia cibagou* Gan, Mi & Wang, sp. nov. A, B, D, F. Female paratype (TRU-XZ-THR-0066) C, E. Male holotype. A. Epigyne, ventral view; B. Vulva, dorsal view; C, D. Habitus, dorsal view; E, F. Ditto, lateral view. Scale bars: 0.1 mm (A, B); 0.5 mm (C–F).

This species is similar to that of *P. septemaculeata* O. Pickard-Cambridge, 1873 in having similar copulatory organs, but it can be easily distinguished by the following: (1) origin of the embolus (E) after the embolic base is directed towards about 1 o’clock in retrolateral view (Fig. 6C) vs towards the 4 o’clock (Nafin et al. 2019: fig. 3A); (2) end of the prolateral tegular apophysis (PTA) is blunt and flat (Fig. 6A) vs sharp and slender (Nafin et al. 2019: fig. 4B); (3) copulatory ducts (CD) are forming a circle on proximal 1/3, and extending beyond the anterior-most level of the spermathecae (Figs 5C, D, 7B) vs not forming similar circle, and just extending to the median level of the spermathecae (Nafin et al. 2019: fig. 4F); and (4) female abdomen is about trapeziform (Fig. 7D) vs quadrate (Nafin et al. 2019: fig. 2A).

##### Description.

**Male** (holotype; Figs 6, 7C, E). Total length 2.75. Carapace 1.07 long, 1.03 wide. Abdomen 2.00 long, 1.95 wide. Carapace yellow brown except edge brown; ocular area protrudes more than female and extends forward to upper part of forehead. Chelicerae, endites and sternum yellowish-brown, labium brown, fused with sternum, endites, sternum and labium covered with brown setae. Legs overall yellow except femora, patellae, tibiae I and tibiae IV dark brown, and metatarsi I yellow brown, tibiae I with 5 short spines. Measurements of legs: I 4.37 (1.63, 1.18, 0.88, 0.68), II 2.86 (1.05, 0.88, 0.40, 0.53), III 2.32 (0.75, 0.73, 0.36, 0.48), IV 3.66 (1.40, 1.16, 0.49, 0.61). Abdomen sclerotized, dorsum yellow, covered with circular impressed dots, with eleven symmetrically arranged, circular orange-brown spots, four small and seven larger and edge with seven short and stout spines; later sub-triangular, with five circular orange-brown spots, and brown sclerotized ring encircled spinnarets; venter colored as dorsum.

***Palp*** (Fig. 6A–C): femur yellow, c. 6 times longer than wide; patella and tibia almost equal in length; tibia gradually widened from base to distal end, with several dorso-distal setae; cymbium ~2 times longer than wide, with tapered antero-retrolateral hook with blunt tip; embolus (E) slender, originates from upper of retrolateral side of bulb, curved into C-shape at origin, and then extending along edge of bulb to top of conductor; embolic base (EB) sub-oval, with short, spiny, baso-prolateral apophysis; conductor (C) membranous and transparent; prolateral tegular apophysis (PTA) flat and elongated, about one-third cymbial length, end truncated.

**Female** (TRU-XZ-THR-0066; Figs 5C, D, 7A, B, D, F). Total length 3.52. Carapace 1.41 long, 1.16 wide. Abdomen 3.03 long, 5.42 wide. Measurements of legs: I 5.10 (2.00, 1.45, 0.81, 0.84), II 3.24 (1.24, 1.00, 0.40, 0.60), III 3.03 (1.08, 1.00, 0.40, 0.55), IV 4.97 (1.86, 1.68, 0.65, 0.78). Habitus (Fig. 7D, F) generally similar to that of male except bulge and protrusion of head region smaller, femora, patellae I dark brown, tibiae I without spines, abdomen sliver, and more swollen.

***Epigyne*** (Figs 5C, D, 7A, B): highly sclerotized, except part of anteromedian portion transparent, with sheet-shaped basal plate (BP) beyond epigastric groove; copulatory openings (CO) small, anterior to basal epigynal plate; copulatory ducts (CD) long, anteroprolaterally extending at origin until forming circle at proximal 1/3, then extending into invert U-shape, and distal end connected to bottom of spermathecae; spermathecae (S) pear-shaped, separated from each other ~1/4 their width; fertilization ducts (FD) tilted upwards.

##### Distribution.

**Figure 8.**
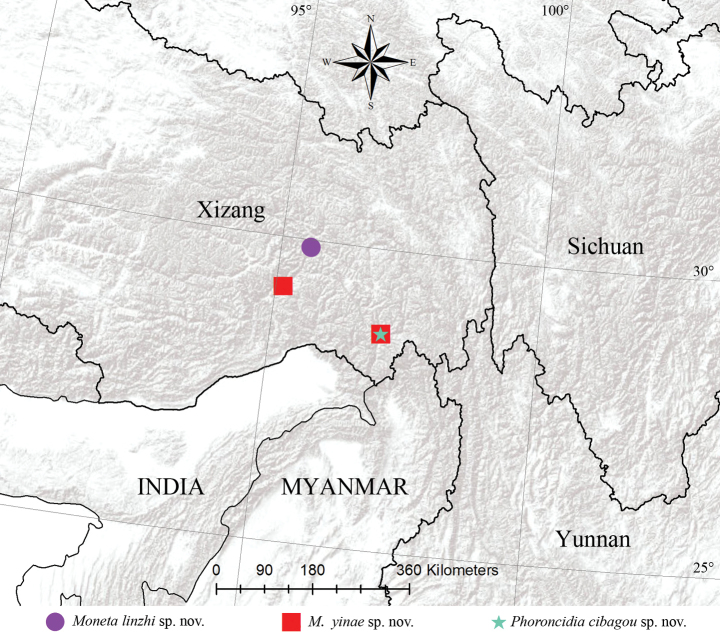
Distributional records of the three new theridiid species.

Known only from the type locality in Xizang, China (Fig. 8).

## Supplementary Material

XML Treatment for Moneta

XML Treatment for Moneta linzhi

XML Treatment for Moneta yinae

XML Treatment for Phoroncidia

XML Treatment for Phoroncidia cibagou

## ﻿Additional information

### Use of AI

No use of AI was reported.

### Author ORCIDs

Jiahui Gan https://orcid.org/0000-0001-6899-3705

Xiaoqi Mi https://orcid.org/0000-0003-1744-3855

Cheng Wang https://orcid.org/0000-0003-1831-0579

Liqing Fan https://orcid.org/0000-0002-8397-741X

### Data availability

All of the data that support the findings of this study are available in the main text.

## References

[B1] Gao CX Li SQ (2014) Comb-footed spiders (Araneae: Theridiidae) in the tropical rainforest of Xishuangbanna, Southwest China. Zoological Systematics 39(1): 1–135.

[B2] Hu JL (2001) Spiders in Qinghai-Tibet Plateau of China. Henan Science and Technology Publishing House, 658 pp.

[B3] Lin YJ Li SQ Mo HL Wang XH (2024a) Thirty-eight spider species (Arachnida: Araneae) from China, Indonesia, Japan and Vietnam. Zoological Systematics 49(1): 4–98. 10.11865/zs.2024101

[B4] Lin YJ Hu CH Pham DS Li SQ (2024b) Taxonomic notes of theridiid spiders (Araneae: Theridiidae) from China and Vietnam. Zoological Research. Diversity and Conservation 1(2): 141–168. 10.24272/j.issn.2097-3772.2024.604

[B5] Nafin KS Sumesh NV Sudhin PP Sudhikumar AV (2019) Redescription and new records of *Phoroncidia septemaculeata* O. Pickard-Cambridge 1873 from India (Araneae: Theridiidae). Zootaxa 4691(2): 188–194. 10.11646/zootaxa.4691.2.9 31719408

[B6] Vanuytven H (2021) The Theridiidae of the World. A key to the genera with their diagnosis and a study of the body length of all known species. Newsletter of the Belgian arachnological. Society 35(Supplement): 1–363.

[B7] WSC [World Spider Catalog] (2025) World Spider Catalog, Version 26. Natural History Museum Bern. 10.24436/2 [Accessed on: 28 July 2025]

[B8] Yamasaki T Hashimoto Y Endo T Hyodo F Itioka T Meleng P (2018) New species of the ant-mimicking genus *Myrmarachne* MacLeay, 1839 (Araneae: Salticidae) from Sarawak, Borneo. Zootaxa 4521(3): 335–356. 10.11646/zootaxa.4521.3.2 30486151

[B9] Zhu MS (1998) Fauna Sinica: Arachnida: Araneae: Theridiidae. Science Press, Beijing, 436 pp.

